# Inter-Basin Water Transfer Supply Chain Coordination with Ramsey Pricing

**DOI:** 10.3390/ijerph16193651

**Published:** 2019-09-28

**Authors:** Zhisong Chen, Keith C.K. Cheung, Manyi Tan

**Affiliations:** 1 Business School, Nanjing Normal University, Qixia District, Nanjing 210023, China; zhisongchen@gmail.com or; 2 Stern School of Business, New York University, 44 West Fourth Street, New York, NY 10012, USA; 3 Odette School of Business, University of Windsor, Windsor, ON N9B 3P4, Canada; cckeung@uwindsor.ca; 4 College of Management Science, Chengdu University of Technology, Chengdu 610059, China

**Keywords:** inter-basin water transfer (IBWT), supply chain, Ramsey pricing, coordination, social welfare maximization, water delivery loss

## Abstract

Often enough, social welfare and private benefit do not align for quasi-public goods/services. The inter-basin water transfer (IBWT) project provides a vivid example of this. In this paper, following the game-theoretical approach, we derive an optimal Ramsey pricing scheme to resolve these conflicts. We try to compare traditional supply chain management models with an optimal Ramsey pricing scheme, with an enforcement of coordination among firms. Using simulation techniques, we compute numerical estimates under three regimes: a standard equilibrium decision framework, a coordination decision model and a coordinated Ramsey pricing scheme. Our results show the relative welfare impact of different settings, revealing that the optimal pricing scheme based on the two-part tariff structure cannot only improve social welfare, but also ensure a target profit for participating firms. Lastly, our findings have strong policy implications for the government with profit regulation and the control of water resources.

## 1. Introduction

The inter-basin water transfer (IBWT) project involves using artificial methods to divert water from the surplus areas to the deficit regions in order to promote the overall economic and social development of a country. Famous examples in the world include the Central Valley Project (CVP) in the United States [1,2], the Tagus-Segura Water Transfer (TSWT) project in Europe [2], and the South-to-North Water Diversion (SNWD) project in China [3]. The Central Valley Project (CVP) is a federal water management project designed, constructed and operated to regulate, store, and transfer water from reservoirs in the Northern half of California to provide irrigation and municipal water to much of the Central Valley, San Joaquin Valley, and its surroundings. The Tagus-Segura Water Transfer (TSWT) project is a Spanish hydraulic engineering design, constructed and operated to transfer water from the Tagus River through the reservoirs of Entrepeñas and Buendía into the Talave Reservoir on the tributary of the Segura River in Spain. The SNWD Project is a multi-decade infrastructure mega-project aiming to transfer 44.8 billion cubic meters of fresh water annually from the Yangtze River in Southern China to the more arid and industrialized North through three canal systems. 

While water itself is a quasi-public good, its delivery service involves public interests from different stakeholders. Thus, IBWT projects have quasi-public-welfare characteristics: the operations of an IBWT project are inseparable from the government’s intervention and regulation. For example, the CVP is operated under the supervision of the United States Bureau of Reclamation (USBR), the TSWT project is operated under the supervision of Spain Ministry for the Ecological Transition, and the SNWD project is operated under the supervision of the China Ministry of Water Resources. Therefore, IBWT projects should take both private and social welfare into account. Unfortunately, operators typically focus only on profit maximization if there is no government intervention. Then, government regulations such as controls on price, quantity, and quality are critical to keep up the social welfare. Ramsey pricing is believed to offer a suitable perspective for solving the problem. Its idea is to set prices to maximize social welfare subject to a profit constraint [4]. Note that this profit constraint can be a guaranteed target level or simply a breakeven point. 

When price distortion from the marginal cost is unavoidable, Ramsey pricing provides a second-best solution [4]. Various kinds of quasi-public goods/services have been priced by governments using the Ramsey scheme to ensure social welfare maximization. The chosen price exceeds the marginal cost by an amount inversely proportional to the elasticity of demand. Although studies about Ramsey pricing are abundant, its application to IBWT projects is sparse. The existing literature has not explored the strategic aspects of IBWT from a supply chain management perspective. Only a few papers can be found pursuing different directions such as the interactive behavior [5] and the bargaining strength [6,7] among players. 

Among the unexplored issues with the Ramsey pricing scheme, we would like to fill the gap by modelling the problem in the framework of coordination games. Our contribution of this paper is to investigate and establish the equilibrium and coordination strategies for an IBWT supply chain with/without Ramsey pricing. We find that an optimal Ramsey pricing scheme through coordination should be pursued to secure those “inherent advantages”. The results are robust.

This paper is organized as follows. Section 2 is the literature review. Section 3 defines the assumptions and notations for a generic IBWT supply chain model. Section 4 examines the impact of Ramsey pricing under three different scenarios applied to the basic IBWT model. Section 5 validates the results with numerical simulation methods. Section 6 discusses the management insights and policy implications. Section 7 concludes with future research.

## 2. Literature Review

To maximize social welfare for quasi-public goods/services, Ramsey theory is taken as an optimal pricing scheme for regulated firms with market influence. Conventional applications of Ramsey pricing are well observed. Examples include studies on airports, railroads, ferries, electricity utility industries, health care sectors, pollution controls, and water resource. Morrison (1982), Martin-Cejas (1997), and Zhang et al. (1997) have developed theoretical models to show that Ramsey prices are optimal with cost recovery problems for the uncongested airports [8,9,10,11]. A similar exercise has been conducted by Jorgensen et al. (1983) for ferries [12]. Other researchers like Tye et al. (1983) argue that Ramsey prices are inefficient for busy hubs when they study railway operations [13]. For the electricity utility industries, Matsukawa et al. (1993) have used the estimated costs and demands to test the impact of Ramsey pricing in Japan [14]. Berry (2000) studied Ramsey pricing as applied to a stranded cost problem in the US power market on the exit decision [15]. In the health care sector, Wedig (1993) constructed a theory of Ramsey pricing for the physician markets to minimize the deadweight loss of oversupply [16]. Ploeg et al. (1991) have developed Ramsey models with the flow externalities, the flow and stock externalities, and the abatement activities in the pollution control [17]. For water resources, Garcia et al. (2002) proposed an econometric model testing Ramsey prices with various technology and demand parameters [18]. Diakite et al. (2009) built an optimal non-linear tariff on Ramsey pricing with heterogenous consumers [19]. Saglam (2012) designed a test showing how a Ramsey price changes the crop composition [20]. 

Research regarding Ramsey pricing has extended along different technical dimensions. Brock et al. (1985) carried out a dynamic Ramsey pricing analysis under an intertemporal framework and found a strong correlation between marginal and average costs and adjustment costs when spill-over happens [21]. Oum et al. (1988) derived a Ramsey pricing rule in the presence of externality costs [22]. Wilson (1989) employed a two-part non-linear tariff structure for the priority service with the Ramsey pricing [23]. The welfare consequences under Ramsey pricing are compared by Dierker (1991), who examines a pure monopoly, and Prieger (1996) who looks at a duopoly [24,25].

A new area of research in the context of Ramsey pricing emerges from the monopolist setting. The IBWT projects in China provide excellent opportunities for empirical tests. Especially, the SNWD initiative has inspired a lot of discussions under the lens of game-theory. Researchers may find interesting exercises to integrate the strategic behavior of the players with the Ramsey pricing models. However, the existing literature has rarely touched upon Ramsey pricing in the IBWT supply chain. Currently, the theories, methods and techniques of supply chain management (SCM) have been applied to the study of the operations management of IBWT projects (especially the SNWD). Ballestero (2004) proposed a decision stochastic approach to determine quantity and price by simulating the recipient’s demand curve and the donor’s supply curve for transferable water [26]. Wang et al. (2012) studied the pricing and coordinating schemes for IBWT supply chain management [5]. Chen et al. (2012a) developed a model with centralization/decentralization decision-making to investigate the coordination mechanism via revenue-sharing [27]. Furthermore, Chen et al. (2012b) used game-theoretical approaches, such as the Stackelberg leadership and Nash bargaining, in studying the supply chain [28]. Moving along the same line, Xu et al. (2012) adopted a finite-horizon periodic review inventory model [29]. Chen et al. (2013) found a two-tier pricing scheme with a Stackelberg leader can integrate government control and market power to maximize both the public and private interests [6]. More recently, Du et al. (2016) compared the impacts of competition intensity on the profits of two water distributors and the water supply chain system under two-part pricing contracts versus wholesale price contracts [30]. Cabo and Tidball (2017) presented a two-regime differential game for the water transfer project and defined an imputation distribution procedure (IDP) to share the investment costs during cooperation according to each player’s future benefits [31]. Du et al. (2018) constructed another pricing-game study for competitive water supply chains under different power structures [32]. Du et al. (2019) investigated the optimal pricing and ordering strategies for dual competing water supply chains under three contracts [33]. Chen et al. (2018, 2019) explored a coordinated game with government subsidy to aid social welfare maximization [7,34].

Although the literature regarding Ramsey pricing and the IBWT supply chain exist, none of them explore the strategic operating incentive of the IBWT supply chain under Ramsey pricing. There is also a lack of comparison between operational performance and social welfare among different operation strategies with/without Ramsey pricing in order to determine an optimal policy to maximize social welfare and private benefit. This paper tries to fulfill these urgent needs.

## 3. Theoretical Modeling Notations and Assumptions

**Figure 1:**
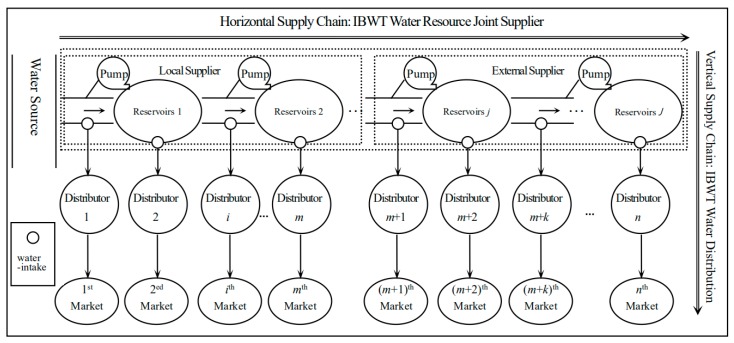
A Generic Inter-Basin Water Transfer Supply Chain System.

An IBWT system can be viewed as an ‘embedded’ supply chain structure, in which a horizontal water supply system is embedded in a vertical water distribution system (see Figure 1). The horizontal supply chain is comprised of a local supplier and an external supplier, and they serve as a joint IBWT supplier via an efficient cooperation mechanism. The vertical supply chain distributes water by the joint IBWT supplier through multiple water distributors to many water consumers in the service region. Specifically, water resources are transferred and supplied by the local supplier from the water source to the external supplier within the trunk channel, and then distributed to water resources distributors of all water-intakes via river channels and artificial canals. Finally, the water resources are sold by each distributor to the water resources consumers in the distributor’s region. According to the actual operating situations of IBWT projects, the IBWT water supply capacity is generally sufficient to meet all downstream demands. Furthermore, what needs to be noted is that water consumers can only buy water from their regional water distributors due to the fixed physical structure of the water transferring channel and the corresponding facilities and equipment. This feature determines that there is no competition among water distributors.

In Figure 1, the water distributors and the corresponding consumers are indexed by i=1,2,…,n. Since the average annual precipitation in an IBWT system is relatively stable (i.e., the extreme climate situation is not considered in this study), its impact on the consumer demands and the IBWT system operations can be neglected in the model. The analytical models developed in this study will focus on the key characteristics of the IBWT supply chain composed of a vertical water distribution system with an embedded horizontal water supply system. We assume there are m distributors supplied by the local supplier and n−m distributors supplied by the external supplier. The water transfer cost from the *i*^th^ water-intake to the *i*^th^ distributor is $c_{di}$, the water transfer cost from the (*k* − 1)^th^ water-intake to the *k*th water-intake within the horizontal green supply chain is $c_{k}$, and the water delivery loss from the (*k* − 1)^th^ water-intake to the *k*th water-intake within the horizontal green supply chain is $\delta_{k}$, k=1,2,…,n. The ordering quantity of the *i*^th^ water-intake (the water demand for the *i*^th^ water distributor) is $q_{i}$, which is delivered from the water source with the original pumping quantity $Q_{i}$. Obviously, the relationship between the water demand of the *i*^th^ water-intake $q_{i}$ and the original pumping quantity $Q_{i}$ is $q_{i}=Q_{i}\prod_{k=1}^{i}\left(1-\delta_{k}\right)$, and the total transfer cost of the original pumping quantity $Q_{i}$ is $TC_{i}\left(Q_{i}\right)=Q_{i}\sum_{k=1}^{i}\left[c_{k}\prod_{j=0}^{k-1}\left(1-\delta_{j}\right)\right]$, hereinto, $\delta_{0}=0$. Therefore, the total transfer cost of the water demand (ordering quantity) of the *i*^th^ water-intake is $TC_{i}\left(q_{i}\right)=\frac{\sum_{k=1}^{i}\left[c_{k}\prod_{j=0}^{k-1}\left(1-\delta_{j}\right)\right]}{\prod_{k=1}^{i}\left(1-\delta_{k}\right)}q_{i}$. Define $C_{i}=\frac{\sum_{k=1}^{i}\left[c_{k}\prod_{j=0}^{k-1}\left(1-\delta_{j}\right)\right]}{\prod_{k=1}^{i}\left(1-\delta_{k}\right)}$, then $TC_{i}\left(q_{i}\right)=C_{i}q_{i}$. The fixed cost for the local supplier is $c_{fl}$, and the fixed cost for the external supplier is $c_{fe}$. Then, the fixed cost for the IBWT supplier is $c_{f}=c_{fl}+c_{fe}$. The local supplier sells and transfers water resources to the external supplier with the wholesale price w (per m^3^). The bargaining power of the local supplier is τ, and the bargaining power of the external supplier is 1−τ, and τ∈(0,1).

A two-part tariff system is often applied in the water pricing for IBWT projects. In Figure 1, the IBWT supplier sells water to the *i*^th^ distributor with a two-part tariff system, i.e., an entry price (a lump-sum fee) $w_{ei}$ and a usage price (charge per-use or per-unit) $w_{i}$. The water demand for the *i*^th^ consumer is $q_{i}$. According to the study of water demand function estimation [35,36], the water demand function for the *i*^th^ water distributor can be expressed in the multiplicative form with an iso-elastic demand curve, i.e., $q_{i}\left(p_{i}\right)=a_{i}p_{i}^{-b}$, where $p_{i}$ is the retail price of water resources for the *i*^th^ water distributor, $a_{i}$ is a positive constant number, which means that the potential maximum water demand quantity and b is the price-elasticity index of the demand. The larger the b value, the more sensitive the demand is to a change in price [37]. The inverse water demand function for the *i*^th^ distributor can be derived as: $p_{i}\left(q_{i}\right)=a_{i}^{\frac{1}{b}}q_{i}^{-\frac{1}{b}}$, i=1,2,…,n.

Based on the foregoing parameter settings and model assumptions, the profit functions of the IBWT supplier, the local supplier, the external supplier, the *i*^th^ water distributor, and the IBWT supply chain are as follows:

(1) $$\Pi_{S}=\sum_{i=1}^{n}\left[\left(w_{i}-C_{i}\right)q_{i}+w_{ei}\right]-c_{f}$$

(2) $$\Pi_{LS}=\sum_{i=1}^{m}\left[\left(w_{i}-C_{i}\right)q_{i}+w_{ei}\right]-c_{fl}+w\sum_{i=m+1}^{n}q_{i}$$

(3) $$\Pi_{ES}=\sum_{i=m+1}^{n}\left[\left(w_{i}-C_{i}\right)q_{i}+w_{ei}\right]-c_{fe}-w\sum_{i=m+1}^{n}q_{i}$$

(4) $$\Pi_{D_{i}}=\left(p_{i}-c_{di}-w_{i}\right)q_{i}-w_{ei},\;i=1,2,\ldots ,n$$

(5) $$\Pi_{SC}=\sum_{i=1}^{n}\left[p_{i}-\left(C_{i}+c_{di}\right)\right]q_{i}-c_{f}$$

According to the concept of economic surplus [38], consumer surplus is the difference between the maximum price a consumer is willing to pay and the actual price they do pay. According to classical economic theory, the total consumer surplus in the IBWT supply chain can be written as [39,40]:

(6) $$CS=\sum_{i=1}^{n}CS_{i}=\sum_{i=1}^{n}\left[\int_{0}^{q_{i}}p_{i}\left(x_{i}\right)dx_{i}-p_{i}q_{i}\right].$$

On this basis, we can express the social welfare function *SW* for the IBWT supply chain as:

(7) $$SW=CS+\Pi_{SC}=\sum_{i=1}^{n}\int_{0}^{q_{i}}p_{i}\left(x_{i}\right)dx_{i}-\sum_{i=1}^{n}\left(C_{i}+c_{di}\right)q_{i}-c_{f}.$$

## 4. IBWT Supply Chain Coordination Decision Model with Ramsey Pricing

Based on the modeling notations and assumptions in Section 3, three game-theoretical decision models, including the benchmark equilibrium decision model without Ramsey pricing, coordination decision model without Ramsey pricing, and the coordination decision model with Ramsey pricing, are developed, analyzed, and compared for the IBWT supply chain in this section.

### 4.1. Benchmark Equilibrium Decision Model without Ramsey Pricing

Under the benchmark equilibrium decision scenario, the detailed decision sequences are as follows: the local supplier and the external supplier will first bargain over the wholesale price w to achieve cooperative operations within the IBWT horizontal supply chain. Then, the IBWT supplier decides all the water usage prices $w_{i}$ simultaneously. Finally, each distributor decides the retail prices $p_{i}$ independently and simultaneously.

#### 4.1.1. IBWT Vertical Supply Chain Equilibrium

In the IBWT vertical supply chain equilibrium model, $w_{ei}=0$. Thus, the optimal problem for the *i*^th^ distributor’s optimal problem is as follows:

(8) $$\underset{p_{i}}{\max}\;\Pi_{D_{i}}=\left(p_{i}-c_{di}-w_{i}\right)q_{i}\left(p_{i}\right),\;i=1,2,\ldots ,n$$

Solving the first-order condition and the second-order derivative of the optimal problem with respect to (w.r.t.) the water retail price $p_{i}$ respectively, and we can obtain the reaction function of the water retail price $p_{i}$ and the ordering quantity $q_{i}$ w.r.t. the water usage price *w_i_* as follows:

(9) $$p_{i}\left(w_{i}\right)=\frac{b}{b-1}\left(c_{di}+w_{i}\right)\;,\;i=1,2,\ldots ,n$$

(10) $$q_{i}\left(w_{i}\right)={\left(\frac{b-1}{b}\right)}^{b}a_{i}{\left(c_{di}+w_{i}\right)}^{-b},\;i=1,2,\ldots ,n$$

Plugging $q_{i}\left(w_{i}\right)$ into the IBWT supplier’s profit function, we can get the optimal problem for the IBWT supplier as follows:

(11) $$\underset{w_{i}}{\max}\;\Pi_{S}=\sum_{i=1}^{n}\left(w_{i}-C_{i}\right)q_{i}\left(w_{i}\right)-c_{f}$$

Solving the first-order condition and the second-order derivative of the optimal problem w.r.t. the water usage price $w_{i}$ respectively, we can obtain the equilibrium water usage price $w_{i}^{b}$ as follows:

(12) $$w_{i}^{b}=\frac{bC_{i}+c_{di}}{b-1},\;i=1,2,\ldots ,n$$

Plugging $w_{i}^{b}$ into $p_{i}\left(w_{i}\right)$ and $q_{i}\left(w_{i}\right)$, we can get the equilibrium retail price $p_{i}^{b}$ and the equilibrium ordering quantity $q_{i}^{b}$ as follows:

(13) $$p_{i}^{b}={\left(\frac{b}{b-1}\right)}^{2}\left(C_{i}+c_{di}\right)\;,\;i=1,2,\ldots ,n$$

(14) $$q_{i}^{b}={\left(\frac{b-1}{b}\right)}^{2b}a_{i}{\left(C_{i}+c_{di}\right)}^{-b},\;i=1,2,\ldots ,n$$

Therefore, the equilibrium profits of the IBWT supplier $\Pi_{S}^{b}$, the distributors $\Pi_{D_{i}}^{b}$, and the IBWT supply chain $\Pi_{SC}^{b}$ are shown below:

(15) $$\Pi_{S}^{b}=\frac{1}{b-1}{\left(\frac{b-1}{b}\right)}^{2b}\sum_{i=1}^{n}a_{i}{\left(C_{i}+c_{di}\right)}^{1-b}-c_{f}$$

(16) $$\Pi_{D_{i}}^{b}=\frac{b}{{\left(b-1\right)}^{2}}{\left(\frac{b-1}{b}\right)}^{2b}a_{i}{\left(C_{i}+c_{di}\right)}^{1-b},\;i=1,2,\ldots ,n$$

(17) $$\Pi_{SC}^{b}=\frac{2b-1}{{\left(b-1\right)}^{2}}{\left(\frac{b-1}{b}\right)}^{2b}\sum_{i=1}^{n}a_{i}{\left(C_{i}+c_{di}\right)}^{1-b}-c_{f}$$

Furthermore, the corresponding consumer surplus and the social welfare are as follows:

(18) $$CS_{b}=\frac{1}{b-1}{\left(\frac{b-1}{b}\right)}^{2\left(b-1\right)}\sum_{i=1}^{n}a_{i}{\left(C_{i}+c_{di}\right)}^{1-b}$$

(19) $$SW_{b}=\left[{\left(\frac{b}{b-1}\right)}^{3}-1\right]{\left(\frac{b-1}{b}\right)}^{2b}\sum_{i=1}^{n}a_{i}{\left(C_{i}+c_{di}\right)}^{1-b}-c_{f}$$

#### 4.1.2. IBWT Horizontal Supply Chain Cooperation 

Plugging $w_{i}^{b}$ and $q_{i}^{b}$ into the profit functions of the local supplier and the external supplier in the IBWT horizontal supply chain, we can get:

(20) $$\Pi_{LS}^{b}\left(w\right)=\frac{1}{b-1}{\left(\frac{b-1}{b}\right)}^{2b}\sum_{i=1}^{m}a_{i}{\left(C_{i}+c_{di}\right)}^{1-b}-c_{fl}+w\sum_{i=m+1}^{n}q_{i}^{b}$$

(21) $$\Pi_{ES}^{b}\left(w\right)=\frac{1}{b-1}{\left(\frac{b-1}{b}\right)}^{2b}\sum_{i=m+1}^{n}a_{i}{\left(C_{i}+c_{di}\right)}^{1-b}-c_{fe}-w\sum_{i=m+1}^{n}q_{i}^{b}$$

According to the Nash bargaining theory [41,42,43,44], an asymmetric Nash bargaining problem for bargaining over the wholesale price *w* can be expressed as follows:

(22) $$\begin{array}{l} \underset{w}{\max}\;\theta \left(w\right)={\left[\Pi_{LS}^{b}\left(w\right)\right]}^{\tau }{\left[\Pi_{ES}^{b}\left(w\right)\right]}^{1-\tau }\\ s.t.\;\Pi_{LS}^{b}\left(w\right)+\Pi_{ES}^{b}\left(w\right)=\Pi_{S}^{b} \end{array}$$

Solving the first-order condition and the second-order derivative of the optimal problem w.r.t. the wholesale price w respectively, we can obtain the bargaining wholesale price *w_b_* as follows:

(23) $$w_{b}=\frac{W_{b}}{\sum_{i=m+1}^{n}q_{i}^{b}}$$

Hereinto,

$$W_{b}=\tau \left[\frac{1}{b-1}{\left(\frac{b-1}{b}\right)}^{2b}\sum_{i=1}^{n}a_{i}{\left(C_{i}+c_{di}\right)}^{1-b}-c_{f}\right]-\left[\frac{1}{b-1}{\left(\frac{b-1}{b}\right)}^{2b}\sum_{i=1}^{m}a_{i}{\left(C_{i}+c_{di}\right)}^{1-b}-c_{fl}\right]$$

Hence, we can get the bargaining profit of the local supplier and the external supplier in the IBWT horizontal supply chain as follows:

(24) $$\Pi_{LS}^{b}=\tau \Pi_{S}^{b}$$

(25) $$\Pi_{ES}^{b}=\left(1-\tau \right)\Pi_{S}^{b}$$

### 4.2. Coordination Decision Model without Ramsey Pricing

Under the coordination decision scenario without Ramsey pricing, the detailed decision sequences are as follows. The local supplier and the external supplier will first bargain over the wholesale price w to achieve cooperative operations within IBWT horizontal supply chain. Then, the IBWT supplier offers the distributors a two-part tariff contract: the IBWT supplier will make the water usage prices $w_{i}$ at the actual transfer cost $C_{i}$ and charge the *i*^th^ water distributor an entry price $w_{ei}$ in return, and the water distributors will make their retail prices $p_{i}$ in accordance with the centralized pricing decision.

#### 4.2.1. Optimal Pricing for the Centralized IBWT Supply Chain

The optimal pricing problem for the centralized IBWT supply chain can be formulated as follows:

(26) $$\underset{p_{i}}{\max}\;\Pi_{SC}\left(p_{i}\right)=\sum_{i=1}^{n}\left[p_{i}-\left(C_{i}+c_{di}\right)\right]q_{i}\left(p_{i}\right)-c_{f}$$

Solving the first-order condition and the second-order derivative of the optimal problem, we can obtain the optimal retail price of the water resources for the *i*^th^ water distributor as follows:

(27) $$p_{i}^{c}=\frac{b}{b-1}\left(C_{i}+c_{di}\right),\;i=1,2,\ldots ,n$$

Thus, the optimal ordering quantity of the water resources for the *i*^th^ water distributor is as follows:

(28) $$q_{i}^{c}={\left(\frac{b-1}{b}\right)}^{b}a_{i}{\left(C_{i}+c_{di}\right)}^{-b},\;i=1,2,\ldots ,n$$

Plugging the optimal retail price and the optimal ordering quantity of the water resources into the profit function of the IBWT supply chain, we can obtain the target optimal profit of the IBWT supply chain as follows:

(29) $$\Pi_{SC}^{c}=\frac{1}{b-1}{\left(\frac{b-1}{b}\right)}^{b}\sum_{i=1}^{n}a_{i}{\left(C_{i}+c_{di}\right)}^{1-b}-c_{f}$$

Furthermore, the corresponding consumer surplus and the social welfare are as follows:

(30) $$CS_{c}=\frac{1}{b-1}{\left(\frac{b-1}{b}\right)}^{b-1}\sum_{i=1}^{n}a_{i}{\left(C_{i}+c_{di}\right)}^{1-b}$$

(31) $$SW_{c}=\frac{2b-1}{{\left(b-1\right)}^{2}}{\left(\frac{b-1}{b}\right)}^{b}\sum_{i=1}^{n}a_{i}{\left(C_{i}+c_{di}\right)}^{1-b}-c_{f}$$

#### 4.2.2. IBWT Vertical Supply Chain Coordination

In the IBWT vertical supply chain coordination model, the IBWT supplier offers the distributors a two-part tariff contract in which the IBWT supplier charges a usage price $w_{i}$ from the *i*^th^ distributor. The distributors either accept or reject the contract. If the distributors accept, they have to pay an entry price $w_{ei}$ to the IBWT supplier, which are determined by the negotiation between the IBWT supplier and distributors. Under the two-part tariff contract, the *i*^th^ distributor’s optimal problem is formulated as follows:

(32) $$\underset{p_{i}}{\max}\;\Pi_{D_{i}}=\left(p_{i}-c_{di}-w_{i}\right)q_{i}\left(p_{i}\right)-w_{ei},\;i=1,2,\ldots ,n$$

Solving the first-order condition and the second-order derivative of the optimal problem w.r.t. the water retail price $p_{i}$ respectively, and we can obtain the reaction function of the water retail price $p_{i}$ w.r.t. the water usage price *w_i_* under the two-part tariff contract as follows:

(33) $$p_{i}^{ct}\left(w_{i}\right)=\frac{b}{b-1}\left(c_{di}+w_{i}\right)\;,\;i=1,2,\ldots ,n$$

Under the two-part tariff contract, to achieve the IBWT supply chain coordination, it is necessary to achieve the coordinated condition: $p_{i}^{c}=p_{i}^{ct}\left(w_{i}\right)$. Then, we have the coordinated wholesale price for the *i*^th^ water-intake of the IBWT supplier as follows:

(34) $$w_{i}^{c}=C_{i},\;i=1,2,\ldots ,n$$

Plugging $w_{i}^{c}$ into the profit functions of the IBWT supplier and the *i*^th^ distributor, we can get $\Pi_{S}^{c}\left(w_{ei}\right)=\sum_{i=1}^{n}w_{ei}-c_{f}$ and $\Pi_{D_{i}}^{c}\left(w_{ei}\right)=\frac{1}{b-1}{\left(\frac{b-1}{b}\right)}^{b}a_{i}{\left(C_{i}+c_{di}\right)}^{1-b}-w_{ei}$.

Therefore, only when the following conditions hold: $\Pi_{S}^{c}\left(w_{ei}\right)\geq \Pi_{S}^{b}$, $\Pi_{D_{i}}^{c}\left(w_{ei}\right)\geq \Pi_{D_{i}}^{b}$, would the IBWT supply chain members have the economic motivation to coordinate—that is, the reasonable interval of the entry price is: $w_{ei}^{c}\in \left[\underline{w_{ei}^{c}},\bar{w_{ei}^{c}}\right],\;i=1,2,\ldots ,n$.

Hereinto,

$$\underline{w_{ei}^{c}}=\frac{1}{b-1}{\left(\frac{b-1}{b}\right)}^{2b}a_{i}{\left(C_{i}+c_{di}\right)}^{1-b}\;\bar{w_{ei}^{c}}=\left[{\left(\frac{b}{b-1}\right)}^{b}-\frac{b}{b-1}\right]\frac{1}{b-1}{\left(\frac{b-1}{b}\right)}^{2b}a_{i}{\left(C_{i}+c_{di}\right)}^{1-b},\;i=1,2,\ldots ,n$$

#### 4.2.3. IBWT Horizontal Supply Chain Cooperation 

Plugging $w_{i}^{c}$, $w_{ei}^{c}$ and $q_{i}^{c}$ into the profit functions of the local supplier and the external supplier in the IBWT horizontal supply chain, we can get:

(35) $$\Pi_{LS}^{c}\left(w\right)=\sum_{i=1}^{m}w_{ei}^{c}-c_{fl}+w\sum_{i=m+1}^{n}q_{i}^{c}$$

(36) $$\Pi_{ES}^{c}\left(w\right)=\sum_{i=m+1}^{n}w_{ei}^{c}-c_{fe}-w\sum_{i=m+1}^{n}q_{i}^{c}$$

The asymmetric Nash bargaining problem for bargaining over the wholesale price *w* can be expressed as follows:

(37) $$\begin{array}{l} \underset{w}{\max}\;\theta \left(w\right)={\left[\Pi_{LS}^{c}\left(w\right)\right]}^{\tau }{\left[\Pi_{ES}^{c}\left(w\right)\right]}^{1-\tau }\\ s.t.\;\Pi_{LS}^{c}\left(w\right)+\Pi_{ES}^{c}\left(w\right)=\Pi_{S}^{c} \end{array}$$

Solving the first-order condition and the second-order derivative of the optimal problem w.r.t. the wholesale price w respectively, we can obtain the bargaining wholesale price $w_{c}$ as follows:

(38) $$w_{c}=\frac{W_{c}}{\sum_{i=m+1}^{n}q_{i}^{c}}$$

Hereinto,

$$W_{c}=\tau \left(\sum_{i=1}^{n}w_{ei}^{c}-c_{f}\right)-\left(\sum_{i=1}^{m}w_{ei}^{c}-c_{fl}\right)$$

Therefore, the coordinated profit of the IBWT supplier $\Pi_{S}^{c}$ and the distributors $\Pi_{D_{i}}^{c}$ under the two-part tariff contract are shown below:

(39) $$\Pi_{S}^{c}=\sum_{i=1}^{n}w_{ei}^{c}-c_{f}$$

(40) $$\Pi_{D_{i}}^{c}=\frac{1}{b-1}{\left(\frac{b-1}{b}\right)}^{b}a_{i}{\left(C_{i}+c_{di}\right)}^{1-b}-w_{ei}^{c},\;i=1,2,\ldots ,n$$

On this basis, we can get the bargaining profit of the local supplier and the external supplier in the IBWT horizontal supply chain as follows:

(41) $$\Pi_{LS}^{c}=\tau \Pi_{S}^{c}$$

(42) $$\Pi_{ES}^{c}=\left(1-\tau \right)\Pi_{S}^{c}$$

### 4.3. Coordination Decision Model with Ramsey Pricing

Under the coordination decision scenario with Ramsey pricing, the detailed decision sequences are as follows. The local supplier and the external supplier will first bargain over the wholesale price w to achieve cooperative operations within IBWT horizontal supply chain. Then, the IBWT supplier offers the distributors a two-part tariff contract: the IBWT supplier will make the water usage prices $w_{i}$ at the actual transfer cost $C_{i}$ and charge the *i*^th^ water distributor an entry price $w_{ei}$ in return, and the water distributors will make their retail prices $p_{i}$ in accordance with the government’s Ramsey pricing decision.

#### 4.3.1. Ramsey Pricing for the IBWT Supply Chain

According to Ramsey pricing theory, the optimal pricing problem for the IBWT supply chain can be formulated as follows:

(43) $$\begin{array}{l} \underset{q_{1},q_{2},\ldots ,q_{n}}{\max}\;SW=\sum_{i=1}^{n}\int_{0}^{q_{i}}p_{i}\left(x_{i}\right)dx_{i}-\sum_{i=1}^{n}\left(C_{i}+c_{di}\right)q_{i}-c_{f}\\ s.t.\;\Pi_{SC}=\sum_{i=1}^{n}\left[p_{i}\left(q_{i}\right)-\left(C_{i}+c_{di}\right)\right]q_{i}-c_{f}=T \end{array}$$

Hereinto, T is the target profit of the IBWT supply chain set via Ramsey pricing by the government.

Constructing and solving the Lagrange function of the optimal problem, we can obtain the optimal retail price of the water resources for the *i*^th^ water distributor under Ramsey pricing rule as follows:

(44) $$p_{i}^{r}=\frac{b}{b-r}\left(C_{i}+c_{di}\right),\;i=1,2,\ldots ,n$$

Hereinto, $r=\frac{\lambda }{1+\lambda }$ is the Ramsey number, r∈(0,1), λ is the Lagrange multiplier.

Thus, the optimal ordering quantity of the water resources for the *i*^th^ water distributor is as follows:

(45) $$q_{i}^{r}={\left(\frac{b-r}{b}\right)}^{b}a_{i}{\left(C_{i}+c_{di}\right)}^{-b},\;i=1,2,\ldots ,n$$

Plugging the optimal retail price and the optimal ordering quantity of the water resources into the profit function of the IBWT supply chain, we can obtain the target optimal profit of the IBWT supply chain as follows:

(46) $$\Pi_{SC}^{r}=\frac{r}{b-r}{\left(\frac{b-r}{b}\right)}^{b}\sum_{i=1}^{n}a_{i}{\left(C_{i}+c_{di}\right)}^{1-b}-c_{f}=T^{*}$$

Furthermore, the corresponding consumer surplus and the social welfare are as follows:

(47) $$CS_{r}=\frac{1}{b-1}{\left(\frac{b-r}{b}\right)}^{b-1}\sum_{i=1}^{n}a_{i}{\left(C_{i}+c_{di}\right)}^{1-b}$$

(48) $$SW_{r}=\frac{b+\left(b-1\right)r}{\left(b-1\right)\left(b-r\right)}{\left(\frac{b-r}{b}\right)}^{b}\sum_{i=1}^{n}a_{i}{\left(C_{i}+c_{di}\right)}^{1-b}-c_{f}$$

#### 4.3.2. IBWT Vertical Supply Chain Coordination

In the IBWT vertical supply chain coordination model, the IBWT supplier offers the distributors a two-part tariff contract in which the IBWT supplier charges a usage price $w_{i}$ from the *i*^th^ distributor. The distributors either accept or reject the contract. If the distributors accept, they have to pay an entry price $w_{ei}$ to the IBWT supplier, which are determined by the negotiation between the IBWT supplier and distributors. Under the two-part tariff contract, the *i*^th^ distributor’s optimal problem is formulated as follows:

(49) $$\underset{p_{i}}{\max}\;\Pi_{D_{i}}=\left(p_{i}-c_{di}-w_{i}\right)q_{i}\left(p_{i}\right)-w_{ei}\;,\;i=1,2,\ldots ,n$$

Solving the first-order condition and the second-order derivative of the optimal problem w.r.t. the water retail price $p_{i}$ respectively, and we can obtain the reaction function of the water retail price $p_{i}$ w.r.t. the water usage price *w_i_* as follows:

(50) $$p_{i}^{rt}\left(w_{i}\right)=\frac{b}{b-1}\left(c_{di}+w_{i}\right),\;i=1,2,\ldots ,n$$

Under the two-part tariff contract, to achieve the IBWT supply chain coordination, it is necessary to achieve the coordinated condition: $p_{i}^{r}=p_{i}^{rt}\left(w_{i}\right)$. Then, we have the coordinated wholesale price for the *i*^th^ water-intake of the IBWT supplier as follows:

(51) $$w_{i}^{r}=\frac{b-1}{b-r}\left(C_{i}+c_{di}\right)-c_{di},\;i=1,2,\ldots ,n$$

Plugging $w_{i}^{r}$ into the profit functions of the IBWT supplier and the *i*^th^ distributor, we can get $\Pi_{S}^{r}\left(w_{ei}\right)=\sum_{i=1}^{n}\left[w_{ei}-\frac{1-r}{b-r}{\left(\frac{b-r}{b}\right)}^{b}a_{i}{\left(C_{i}+c_{di}\right)}^{1-b}\right]-c_{f}$ and $\Pi_{D_{i}}^{r}\left(w_{ei}\right)=\frac{1}{b-r}{\left(\frac{b-r}{b}\right)}^{b}a_{i}{\left(C_{i}+c_{di}\right)}^{1-b}-w_{ei}$.

Therefore, only when the following conditions hold: $\Pi_{S}^{r}\left(w_{ei}\right)\geq \Pi_{S}^{b}$, $\Pi_{D_{i}}^{r}\left(w_{ei}\right)\geq \Pi_{D_{i}}^{b}$, would the IBWT supply chain members have the economic motivation to coordinate under Ramsey pricing—that is, the reasonable interval of the entry price is: $w_{ei}^{r}\in \left[\underline{w_{ei}^{r}},\bar{w_{ei}^{r}}\right],\;i=1,2,\ldots ,n$.

Hereinto,

$$\underline{w_{ei}^{r}}=\left[\frac{1}{b-1}{\left(\frac{b-1}{b}\right)}^{2b}+\frac{1-r}{b-r}{\left(\frac{b-r}{b}\right)}^{b}\right]a_{i}{\left(C_{i}+c_{di}\right)}^{1-b}\;\bar{w_{ei}^{r}}=\left[\frac{1}{b-r}{\left(\frac{b-r}{b}\right)}^{b}-\frac{b}{{\left(b-1\right)}^{2}}{\left(\frac{b-1}{b}\right)}^{2b}\right]a_{i}{\left(C_{i}+c_{di}\right)}^{1-b},\;i=1,2,\ldots ,n.$$

#### 4.3.3. IBWT Horizontal Supply Chain Cooperation 

Plugging $w_{i}^{r}$ and $q_{i}^{r}$ into the profit functions of the local supplier and the external supplier in the IBWT horizontal supply chain, we can get:

(52) $$\Pi_{LS}^{r}\left(w\right)=\sum_{i=1}^{m}\left[w_{ei}^{r}-\frac{1-r}{b-r}\left(C_{i}+c_{di}\right)q_{i}^{r}\right]-c_{fl}+w\sum_{i=m+1}^{n}q_{i}^{r}$$

(53) $$\Pi_{ES}^{r}\left(w\right)=\sum_{i=m+1}^{n}\left[w_{ei}^{r}-\frac{1-r}{b-r}\left(C_{i}+c_{di}\right)q_{i}^{r}\right]-c_{fe}-w\sum_{i=m+1}^{n}q_{i}^{r}$$

The asymmetric Nash bargaining problem for bargaining over the wholesale price *w* can be expressed as follows:

(54) $$\begin{array}{l} \underset{w}{\max}\;\theta \left(w\right)={\left[\Pi_{LS}^{r}\left(w\right)\right]}^{\tau }{\left[\Pi_{ES}^{r}\left(w\right)\right]}^{1-\tau }\\ s.t.\;\Pi_{LS}^{r}\left(w\right)+\Pi_{ES}^{r}\left(w\right)=\Pi_{S}^{r} \end{array}$$

Hereinto, τ is the bargaining power of the local supplier. 

Solving the first-order condition and the second-order derivative of the optimal problem w.r.t. the wholesale price w respectively, we can obtain the bargaining wholesale price *w_r_* as follows:

(55) $$w_{r}=\frac{W_{r}}{\sum_{i=m+1}^{n}q_{i}^{r}}$$

Hereinto,

$$W_{r}=\tau \left\{\sum_{i=1}^{n}\left[w_{ei}^{r}-\frac{1-r}{b-r}\left(C_{i}+c_{di}\right)q_{i}^{r}\right]-c_{f}\right\}-\left\{\sum_{i=1}^{m}\left[w_{ei}^{r}-\frac{1-r}{b-r}\left(C_{i}+c_{di}\right)q_{i}^{r}\right]-c_{fl}\right\}$$

Therefore, the coordinated profit of the IBWT supplier $\Pi_{S}^{r}$ and the distributors $\Pi_{D_{i}}^{r}$ under the two-part tariff contract are shown below:

(56) $$\Pi_{S}^{r}=\sum_{i=1}^{n}\left[w_{ei}^{r}-\frac{1-r}{b-r}{\left(\frac{b-r}{b}\right)}^{b}a_{i}{\left(C_{i}+c_{di}\right)}^{1-b}\right]-c_{f}$$

(57) $$\Pi_{D_{i}}^{r}=\frac{1}{b-r}{\left(\frac{b-r}{b}\right)}^{b}a_{i}{\left(C_{i}+c_{di}\right)}^{1-b}-w_{ei}^{r},\;i=1,2,\ldots ,n$$

On this basis, we can get the bargaining profit of the local supplier and the external supplier in the IBWT horizontal supply chain as follows:

(58) $$\Pi_{LS}^{r}=\tau \Pi_{S}^{r}$$

(59) $$\Pi_{ES}^{r}=\left(1-\tau \right)\Pi_{S}^{r}$$

### 4.4. Analytical Results Comparison 

The analytical results of the IBWT supply chain coordination and cooperation considering the water delivery loss under the scenario without considering/considering Ramsey pricing, including the optimal/equilibrium solutions and the corresponding profits and social welfare, are summarized in Table 1 and compared to derive the optimal pricing policies and operating mechanisms for the IBWT supply chain as follows: 

(1) Comparing the analytical results between the equilibrium decision without Ramsey pricing and the coordination decision without Ramsey pricing, (i) the usage prices of water resources under the coordination decision without Ramsey pricing are lower than those under the equilibrium decision without Ramsey pricing; (ii) the retail prices of water resources under the coordination decision without Ramsey pricing are lower than those under the equilibrium decision without Ramsey pricing; (iii) the ordering quantities of water resources under the coordination decision without Ramsey pricing are higher than those under the equilibrium decision without Ramsey pricing; (iv) the profits of the IBWT supply chain and its members under the coordination decision without Ramsey pricing are higher than those under the equilibrium decision without Ramsey pricing; (v) the consumer surplus under the coordination decision without Ramsey pricing is higher than that under the equilibrium decision without Ramsey pricing; (vi) the social welfare under the coordination decision without Ramsey pricing is higher than that under the equilibrium decision without Ramsey pricing.

(2) Comparing the analytical results between the equilibrium decision without Ramsey pricing and the coordination decision with Ramsey pricing, (i) the usage prices of water resources under the coordination decision with Ramsey pricing are lower than those under the equilibrium decision without Ramsey pricing; (ii) the retail prices of water resources under the coordination decision with Ramsey pricing are lower than those under the equilibrium decision without Ramsey pricing; (iii) the ordering quantities of water resources under the coordination decision with Ramsey pricing are higher than those under the equilibrium decision without Ramsey pricing; (iv) the profits of the IBWT supply chain and its members under the coordination decision with Ramsey pricing are higher than those under the equilibrium decision without Ramsey pricing; (v) the consumer surplus under the coordination decision with Ramsey pricing is higher than that under the equilibrium decision without Ramsey pricing; (vi) the social welfare under the coordination decision with Ramsey pricing is higher than that under the equilibrium decision without Ramsey pricing.

(3) Comparing the analytical results between the coordination decision without Ramsey pricing and the coordination decision with Ramsey pricing, (i) the usage prices of water resources under the coordination decision with Ramsey pricing are lower than those under the coordination decision without Ramsey pricing; (ii) the retail prices of water resources under the coordination decision with Ramsey pricing are lower than those under the coordination decision without Ramsey pricing; (iii) the ordering quantities of water resources under the coordination decision with Ramsey pricing are higher than those under the coordination decision without Ramsey pricing; (iv) the profit of the IBWT supply chain under the coordination decision with Ramsey pricing is lower than that under the coordination decision without Ramsey pricing; (v) the consumer surplus under the coordination decision with Ramsey pricing is higher than that under the coordination decision without Ramsey pricing; (vi) the social welfare under the coordination decision with Ramsey pricing is higher than that under the coordination decision without Ramsey pricing.

(4) No matter whether under the equilibrium decision without Ramsey pricing, or under the coordination decision without/with Ramsey pricing, as the water delivery loss rate increases, the profits of IBWT supply chain and its members, the consumer surplus and the social welfare decreases, respectively.

(5) Under the coordination decision with Ramsey pricing, as the Ramsey coefficient increases, the profits of IBWT supply chain and its members increases, the consumer surplus decreases, and the social welfare decreases. 

## 5. Numerical and Sensitivity Analyses

As this study focuses on exploring the pricing regulation policies and operational strategies for a generic IBWT supply chain, the corresponding numerical and sensitivity analyses are conducted to validate and supplement the foregoing modeling analysis results and derive the general pricing regulation policies and operational strategies for a generic IBWT supply chain. Thus, based on the real characteristics of IBWT projects, e.g., the SNWD project in China (Wang et al., 2009), the supply chain structure, the relationships among stakeholders (including the local supplier, external supplier and distributors), and the values of the parameters and their relationships in the IBWT supply chain are set to mimic the real-world case.

Without any loss of generality, an IBWT supply chain with one local supplier, one external supplier, and six water distributors is developed for numerical analysis. Since there are six water-intakes and six water distributors in the IBWT supply chain, i.e., *n* = 6. We assume that three water distributors are supplied by the local supplier (i.e., *m* = 3) and three water distributors are supplied by the external supplier (i.e., *n − m* = 3). Table 2 and Table 3 list the parameters mainly relating to the IBWT supply chain and their values for the numerical analysis. Generally, the transferring cost increases as the water diversion cascade increases. Thus, the water transferring cost from the (*i* − 1)^th^ water-intake to the *i*^th^ water-intake and the water transferring cost from the *i*^th^ water-intake to *i*^th^ water distributor are roughly set as Table 2, based on the water price of eastern and middle routes of the SNWD project [44,45]. Likewise, the farther away from the water source, the greater the water shortage and the greater the water demand. Thus, the potential maximum water demand quantity is set as Table 2. Without a loss of generality, the delivery loss rate is set at 5%, the price-elasticity index of demand is set at 1.5, the Ramsey coefficient is set at 0.6, and both the local and external supplier’s fixed costs are set at 50,000. Due to the local supplier’s strong market power within the IBWT horizontal supply chain, the local supplier’s bargaining power is set at 0.6.

### 5.1. Numerical Analysis

The numerical analysis assesses and compares the pricing and quantity decisions and the resulting profits of the IBWT supply chain and its members, the corresponding consumer surplus and social welfare for the IBWT supply chain equilibrium, and coordination models considering Ramsey pricing or not. The benchmark numerical analysis results of IBWT supply chain equilibrium is shown in Table 4, and the numerical analysis results of IBWT supply chain coordination without/with Ramsey pricing are shown in Table 5 and Table 6.

The findings from the numerical analysis results are summarized below:

(1) Comparing the numerical analysis results between the equilibrium decision without Ramsey pricing (Table 4) and the coordination decision without Ramsey pricing (Table 5), (i) the usage prices of water resources under the coordination decision without Ramsey pricing are lower than those under the equilibrium decision without Ramsey pricing; (ii) the retail prices of water resources under the coordination decision without Ramsey pricing are lower than those under the equilibrium decision without Ramsey pricing; (iii) the ordering quantities of water resources under the coordination decision without Ramsey pricing are higher than those under the equilibrium decision without Ramsey pricing; (iv) the profit of the IBWT supply chain under the coordination decision without Ramsey pricing is higher than that under the equilibrium decision without Ramsey pricing; (v) the profits of the IBWT local supplier and external supplier under the coordination decision without Ramsey pricing are higher than those under the equilibrium decision without Ramsey pricing; (vi) the profits of the IBWT distributors under the coordination decision without Ramsey pricing are higher than those under the equilibrium decision without Ramsey pricing; (vii) the consumer surplus under the coordination decision without Ramsey pricing is higher than that under the equilibrium decision without Ramsey pricing; (viii) the social welfare under the coordination decision without Ramsey pricing is higher than that under the equilibrium decision without Ramsey pricing.

(2) Comparing the numerical analysis results between the equilibrium decision without Ramsey pricing (Table 4) and the coordination decision with Ramsey pricing (Table 6), (i) the usage prices of water resources under the coordination decision with Ramsey pricing are lower than those under the equilibrium decision without Ramsey pricing; (ii) the retail prices of water resources under the coordination decision with Ramsey pricing are lower than those under the equilibrium decision without Ramsey pricing; (iii) the ordering quantities of water resources under the coordination decision with Ramsey pricing are higher than those under the equilibrium decision without Ramsey pricing are higher than those under the equilibrium decision without Ramsey pricing; (iv) the profit of the IBWT supply chain under the coordination decision with Ramsey pricing is higher than that under the equilibrium decision without Ramsey pricing; (v) the profits of the IBWT local supplier and external supplier under the coordination decision with Ramsey pricing are higher than those under the equilibrium decision without Ramsey pricing; (vi) the profits of the IBWT distributors under the coordination decision with Ramsey pricing are higher than those under the equilibrium decision without Ramsey pricing; (vii) the consumer surplus under the coordination decision with Ramsey pricing is higher than that under the equilibrium decision without Ramsey pricing; (viii) the social welfare under the coordination decision with Ramsey pricing is higher than that under the equilibrium decision without Ramsey pricing.

(3) Comparing the numerical analysis results between the coordination decision without Ramsey pricing (Table 5) and the coordination decision with Ramsey pricing (Table 6), (i) the usage prices of water resources under the coordination decision with Ramsey pricing are lower than those under the coordination decision without Ramsey pricing; (ii) the retail prices of water resources under the coordination decision with Ramsey pricing are lower than those under the coordination decision without Ramsey pricing; (iii) the ordering quantities of water resources under the coordination decision with Ramsey pricing are higher than those under the coordination decision without Ramsey pricing; (iv) the profit of the IBWT supply chain under the coordination decision with Ramsey pricing is lower than that under the coordination decision without Ramsey pricing; (v) the profits of the IBWT local supplier and external supplier under the coordination decision with Ramsey pricing are higher than those under the coordination decision without Ramsey pricing; (vi) the profits of the IBWT distributors under the coordination decision with Ramsey pricing are lower than those under the coordination decision without Ramsey pricing; (vii) the consumer surplus under the coordination decision with Ramsey pricing is higher than that under the coordination decision without Ramsey pricing; (viii) the social welfare under the coordination decision with Ramsey pricing is higher than that under the coordination decision without Ramsey pricing.

### 5.2. Sensitivity Analysis

The sensitivity analysis assesses and compares the impacts of the changes of the water delivery loss rate and Ramsey coefficient on the profits of the IBWT supply chain and its members, the corresponding consumer surplus and social welfare for the IBWT supply chain equilibrium and coordination models considering Ramsey pricing or not.

The sensitivity analysis results of the water delivery loss rate for the IBWT supply chain equilibrium and coordination decision without/with Ramsey pricing are shown in Table 7a–c. The results show that, (1) under the benchmark equilibrium decision without Ramsey pricing (Table 7a), as the water delivery loss rate increases, (i) the profits of IBWT supply chain and its members decrease, (ii) the corresponding consumer surplus decreases, (iii) the corresponding social welfare decreases; (2) under the coordination decision without Ramsey pricing (Table 7b), as the water delivery loss rate increases, (i) the profits of IBWT supply chain decreases, (ii) the profits of water distributors decrease, (iii) the corresponding consumer surplus decreases, and (iv) the corresponding social welfare decreases; (3) under the coordination decision with Ramsey pricing (Table 7c), as the water delivery loss rate increases, (i) the profits of IBWT supply chain decreases, (ii) the profits of water distributors decrease, (iii) the profits of IBWT local supplier and IBWT external supplier increase, (iv) the corresponding consumer surplus decreases, (v) the corresponding social welfare decreases.

The sensitivity analysis results of Ramsey coefficient for the IBWT supply chain coordination models with Ramsey pricing is shown in Table 8. The results show that, as the Ramsey coefficient increases, (i) the profits of IBWT supply chain increases, (ii) the profits of water distributors decrease, (iii) the profits of IBWT local supplier and IBWT external supplier increase, (iv) the corresponding consumer surplus decreases, and (v) the corresponding social welfare decreases.

## 6. Managerial Insights and Policy Implications

According to the modelling and numerical analysis results and research findings of Section 4 and Section 5, the corresponding managerial insights and policy implications can be summarized as follows:

First, the coordination decision outperforms the equilibrium decision regarding the profits of the IBWT supply chain, the consumer surplus and the social welfare under the scenario without the government’s Ramsey pricing. Hence, the coordination strategy based on the two-part tariff contract could effectively coordinate the IBWT supply chain without the government’s Ramsey pricing and improve the profits of the IBWT supply chain members and is recommended for the optimal operation management of the IBWT supply chain.

Second, coordination decision with Ramsey pricing outperforms that without Ramsey pricing regarding the consumer surplus and the social welfare, however, coordination decision without Ramsey pricing outperforms that with Ramsey pricing regarding the profits of the IBWT supply chain and its members. Hence, for the government, it would hope to make pricing decision to achieve social welfare maximization according to Ramsey pricing rule; while, for the IBWT supply chain, they would hope to make pricing decision by themselves to optimize their profits. Owing to the quasi-public-goods characteristics of the water resources and the quasi-public-welfare characteristics of the IBWT projects, the goals of social welfare maximization should be given priority in the operations management of the IBWT supply chain. Therefore, Ramsey pricing regulation is recommended to improve the consumer surplus and the social welfare, and also to guarantee a target profit for the IBWT supply chain and its members in the optimal operations management of IBWT supply chain.

Third, the value of Ramsey coefficient hinges on the extent to which the IBWT supply chain profit constraint is binding. The larger the Ramsey coefficient is, the more profits are gained by the IBWT supply chain and its members, and the less consumer surplus and social welfare are achieved by the government. Therefore, a proper Ramsey coefficient is beneficial for balancing the conflict between the IBWT supply chain profits and the consumer surplus and the social welfare.

Finally, the water delivery loss rate plays an important role on the profits of all stakeholders in the IBWT supply chain, the consumer surplus and the social welfare. The less the water delivery loss rate is, the more profits are gained by the IBWT supply chain and its members, and the bigger consumer surplus and social welfare are achieved by the government. Therefore, reducing the water delivery loss rate could effectively benefit each stakeholder in the IBWT supply chain.

In summary, from the perspective of actual governance and operations, the government should regulate the pricing decision of an IBWT supply chain via Ramsey pricing rule, and an IBWT supply chain would be better off adopting coordination strategy based on two-part tariff contract under the government’s Ramsey pricing. In this way, the consumer surplus and social welfare could be improved. Meanwhile, the IBWT supply chain and its members could achieve the corresponding target profits.

## 7. Conclusions

Owing to the quasi-public-goods characteristics of the water resources and the quasi-public-welfare characteristics of IBWT projects, both social welfare and economic benefits should be taken into account in the operations management of the IBWT supply chain. Ramsey pricing regulation is introduced to the operations management of the IBWT supply chain to achieve social welfare maximization and, in the meantime, guarantee a target profit for the IBWT supply chain. On this basis, a benchmark equilibrium decision model without Ramsey pricing, a coordination decision model without Ramsey pricing and a coordination decision model with Ramsey pricing for the IBWT supply chain are developed, analyzed, and compared through the game-theoretic and coordination research approaches, and the corresponding numerical and sensitivity analysis for all models are conducted and compared. Finally, the corresponding management insights and policy implications are summarized in this article. The research results indicate that: (1) For the IBWT supply chain, the coordination strategy, based on the two-part tariff contract, could effectively coordinate the IBWT supply chain and improve the profits of IBWT supply chain members, no matter whether the government adopts Ramsey pricing regulation or not. (2) Ramsey pricing regulation could effectively improve the consumer surplus and social welfare, but reduce the profits of the IBWT supply chain and its members. Hence, the government would hope to adopt Ramsey pricing regulation to improve the consumer surplus and achieve social welfare maximization, while, the IBWT supply chain would hope to make price of water resources themselves to optimize their profits without government’s pricing regulation. (3) Owing to the quasi-public-goods characteristics of water resources and the quasi-public-welfare characteristics of IBWT projects, the goals of social welfare maximization should be given priority in the operations management of IBWT supply chain. Therefore, for the government, Ramsey pricing regulation is recommended to improve the consumer surplus, achieve social welfare maximization, and guarantee a target profit in the optimal operations management of IBWT supply chain. (4) The value of the Ramsey coefficient depends on the extent to which the IBWT supply chain profit constraint is binding. A proper Ramsey coefficient is beneficial for balancing the conflict between the IBWT supply chain profits and the consumer surplus and the social welfare. (5) Reducing the water delivery loss rate could effectively improve the IBWT supply chain profit, the consumer surplus and the social welfare.

In terms of theoretical work, the existing literature barely covers the pricing regulation policies and operational strategies of the IBWT supply chain. This study provides a novel and useful approach for these issues via Ramsey pricing theory to enhance the optimality for the IBWT projects. In practice, the formulation and numerical analysis provide a solid base for governments to design appropriate pricing and regulatory policies and for IBWT stakeholders to come up with efficient strategies for operations.

With the resource constraints and technical difficulties on empirical data collection, this study can only proceed with a game-theoretical decision model of Ramsey pricing using a simulated real-world numerical and sensitivity analyses. All results are robust enough to derive general pricing regulation policies and operational strategies for a generic IBWT supply chain. Admittedly, there are still many important possible extensions for future research. First, supply capacity constraint and water shortage issues can be addressed. Second, the uncertainty factors of random precipitation, water quality, and water environment can be modeled. Third, other types of coordination contracts, such as revenue sharing contract and options contracts, may also be considered. Fourth, water use efficiency may be explored and compared to investigate the impact of the Ramsey price optimization methodology. Finally, the empirical data may be collected, or the actual parameters may be obtained from a real-world case, in order to conduct the corresponding numerical and sensitivity analyses to show the efficiency gain for each party involved.

## Figures and Tables

**Table 1:** Analytical Results of IBWT Supply Chain Coordination with Ramsey Pricing.

Scenarios	Benchmark Equilibrium without Ramsey Pricing	Coordination without Ramsey Pricing	Coordination with Ramsey Pricing
$w_{i}^{*}$	$w_{i}^{b}=\frac{bC_{i}+c_{di}}{b-1}$	$w_{i}^{c}=C_{i}$	$w_{i}^{r}=\frac{b-1}{b-r}\left(C_{i}+c_{di}\right)-c_{di}$
$p_{i}^{*}$	$p_{i}^{b}={\left(\frac{b}{b-1}\right)}^{2}\left(C_{i}+c_{di}\right)$	$p_{i}^{c}=\frac{b}{b-1}\left(C_{i}+c_{di}\right)$	$p_{i}^{r}=\frac{b}{b-r}\left(C_{i}+c_{di}\right)$
$q_{i}^{*}$	$q_{i}^{b}={\left(\frac{b-1}{b}\right)}^{2b}a_{i}{\left(C_{i}+c_{di}\right)}^{-b}$	$q_{i}^{c}={\left(\frac{b-1}{b}\right)}^{b}a_{i}{\left(C_{i}+c_{di}\right)}^{-b}$	$q_{i}^{r}={\left(\frac{b-r}{b}\right)}^{b}a_{i}{\left(C_{i}+c_{di}\right)}^{-b}$
$w_{*}$	$w_{b}=\frac{W_{b}}{\sum_{i=m+1}^{n}q_{i}^{b}}$	$w_{c}=\frac{W_{c}}{\sum_{i=m+1}^{n}q_{i}^{c}}$	$w_{r}=\frac{W_{r}}{\sum_{i=m+1}^{n}q_{i}^{r}}$
$\Pi_{S}^{*}$	$\Pi_{S}^{b}=\frac{1}{b-1}{\left(\frac{b-1}{b}\right)}^{2b}\sum_{i=1}^{n}a_{i}{\left(C_{i}+c_{di}\right)}^{1-b}-c_{f}$	$\Pi_{S}^{c}=\sum_{i=1}^{n}w_{ei}^{c}-c_{f}$	$\Pi_{S}^{r}=\sum_{i=1}^{n}\left[w_{ei}^{r}-\frac{1-r}{b-r}{\left(\frac{b-r}{b}\right)}^{b}a_{i}{\left(C_{i}+c_{di}\right)}^{1-b}\right]-c_{f}$
$\Pi_{LS}^{*}$	$\Pi_{LS}^{b}=\tau \Pi_{S}^{b}$	$\Pi_{LS}^{c}=\tau \Pi_{S}^{c}$	$\Pi_{LS}^{r}=\tau \Pi_{S}^{r}$
$\Pi_{ES}^{*}$	$\Pi_{ES}^{b}=\left(1-\tau \right)\Pi_{S}^{b}$	$\Pi_{ES}^{c}=\left(1-\tau \right)\Pi_{S}^{c}$	$\Pi_{ES}^{r}=\left(1-\tau \right)\Pi_{S}^{r}$
$\Pi_{D_{i}}^{*}$	$\Pi_{D_{i}}^{b}=\frac{b}{{\left(b-1\right)}^{2}}{\left(\frac{b-1}{b}\right)}^{2b}a_{i}{\left(C_{i}+c_{di}\right)}^{1-b}$	$\Pi_{D_{i}}^{c}=\frac{1}{b-1}{\left(\frac{b-1}{b}\right)}^{b}a_{i}{\left(C_{i}+c_{di}\right)}^{1-b}-w_{ei}^{c}$	$\Pi_{D_{i}}^{r}=\frac{1}{b-r}{\left(\frac{b-r}{b}\right)}^{b}a_{i}{\left(C_{i}+c_{di}\right)}^{1-b}-w_{ei}^{r}$
$\Pi_{SC}^{*}$	$\Pi_{SC}^{b}=\frac{2b-1}{{\left(b-1\right)}^{2}}{\left(\frac{b-1}{b}\right)}^{2b}\sum_{i=1}^{n}a_{i}{\left(C_{i}+c_{di}\right)}^{1-b}-c_{f}$	$\Pi_{SC}^{c}=\frac{1}{b-1}{\left(\frac{b-1}{b}\right)}^{b}\sum_{i=1}^{n}a_{i}{\left(C_{i}+c_{di}\right)}^{1-b}-c_{f}$	$\Pi_{SC}^{r}=\frac{r}{b-r}{\left(\frac{b-r}{b}\right)}^{b}\sum_{i=1}^{n}a_{i}{\left(C_{i}+c_{di}\right)}^{1-b}-c_{f}$
$CS_{*}$	$CS_{b}=\frac{1}{b-1}{\left(\frac{b-1}{b}\right)}^{2\left(b-1\right)}\sum_{i=1}^{n}a_{i}{\left(C_{i}+c_{di}\right)}^{1-b}$	$CS_{c}=\frac{1}{b-1}{\left(\frac{b-1}{b}\right)}^{b-1}\sum_{i=1}^{n}a_{i}{\left(C_{i}+c_{di}\right)}^{1-b}$	$CS_{r}=\frac{1}{b-1}{\left(\frac{b-r}{b}\right)}^{b-1}\sum_{i=1}^{n}a_{i}{\left(C_{i}+c_{di}\right)}^{1-b}$
$SW_{*}$	$SW_{b}=\left[{\left(\frac{b}{b-1}\right)}^{3}-1\right]{\left(\frac{b-1}{b}\right)}^{2b}\sum_{i=1}^{n}a_{i}{\left(C_{i}+c_{di}\right)}^{1-b}-c_{f}$	$SW_{c}=\frac{2b-1}{{\left(b-1\right)}^{2}}{\left(\frac{b-1}{b}\right)}^{b}\sum_{i=1}^{n}a_{i}{\left(C_{i}+c_{di}\right)}^{1-b}-c_{f}$	$SW_{r}=\frac{b+\left(b-1\right)r}{\left(b-1\right)\left(b-r\right)}{\left(\frac{b-r}{b}\right)}^{b}\sum_{i=1}^{n}a_{i}{\left(C_{i}+c_{di}\right)}^{1-b}-c_{f}$
$Range\;of\;w_{ei}^{*}$	-	$w_{ei}^{c}\in \left[\underline{w_{ei}^{c}},\bar{w_{ei}^{c}}\right]$	$w_{ei}^{r}\in \left[\underline{w_{ei}^{r}},\bar{w_{ei}^{r}}\right]$

**Table 2:** Parameters in the IBWT vertical supply chain for the numerical analysis.

Water-Intake i	Water Transferring Cost (*i* − 1)^th^ Water-Intake→ $i^{\mathrm{th}}$ Water-Intake $c_{i}$	Delivery Loss Rate $\delta_{i}$	Actual Water Transferring Cost (*i* − 1)^th^ Water-Intake→ $i^{\mathrm{th}}$ Water-Intake $C_{i}$	Water Transferring Cost *i*^th^ Water-Intake→ $i^{\mathrm{th}}$ Water Distributor $c_{di}$	$\mathrm{Positive}\;\mathrm{Constant}\;a_{i}$
1	0.25	5%	0.26	0.05	50,000,000
2	0.30	5%	0.59	0.06	100,000,000
3	0.35	5%	0.99	0.07	150,000,000
4	0.40	5%	1.47	0.08	200,000,000
5	0.45	5%	2.02	0.09	250,000,000
6	0.50	5%	2.65	0.10	300,000,000

**Table 3:** Parameters in the IBWT horizontal supply chain for the numerical analysis.

Parameter	Title	Value
b	price-elasticity index of demand	1.5
r	Ramsey Coefficient	0.6
$c_{fl}$	local supplier’s fixed cost	50,000
$c_{fe}$	external supplier’s fixed cost	50,000
$c_{f}$	IBWT supplier’s fixed cost (*c_fl_* + *c_fe_*)	100,000
τ	local supplier’s bargaining power	0.6

**Table 4:** Benchmark Numerical Analysis Results of IBWT Supply Chain Equilibrium without Ramsey pricing.

i	$w_{i}^{b}$	$p_{i}^{b}$	$q_{i}^{b}$	$\Pi_{D_{i}}^{b}$	$\Pi_{S}^{b}$
1	0.89	2.82	10,567,231	19,855,271	64,544,476
2	1.90	5.88	7,022,120	27,504,145	$\Pi_{LS}^{b}$
3	3.12	9.56	5,073,223	32,339,320	38,726,686
4	4.56	13.91	3,854,577	35,748,212	$\Pi_{ES}^{b}$
5	6.23	18.96	3,028,479	38,277,505	25,817,790
6	8.15	24.74	2,437,812	40,208,975	$\Pi_{SC}^{b}$
Total	-	-	31,983,442	193,933,428	258,477,904
$w_{b}=1.31$, $SW_{b}=840,278,188$, $CS_{b}=581,800,284$					

**Table 5:** Numerical Analysis Results of IBWT Supply Chain Coordination without Ramsey pricing.

i	$w_{ei}^{c}$	$w_{i}^{c}$	$p_{i}^{c}$	$q_{i}^{c}$	$\Pi_{D_{i}}^{c}$	$\Pi_{S}^{c}$	$Range\;of\;w_{ei}^{c}$
1	7,000,000	0.26	0.94	54,908,942	27,390,337	66,900,000	[6,618,424, 14,535,067]
2	10,000,000	0.59	1.96	36,488,004	37,638,577	$\Pi_{LS}^{c}$	[9,168,048, 20,134,432]
3	11,000,000	0.99	3.19	26,361,238	45,013,346	40,140,000	[10,779,773, 23,674,026]
4	12,000,000	1.47	4.64	20,028,972	49,917,720	$\Pi_{ES}^{c}$	[11,916,071, 26,169,508]
5	13,000,000	2.02	6.32	15,736,441	53,298,584	26,760,000	[12,759,168, 28,021,079]
6	14,000,000	2.65	8.25	12,667,244	55,643,987	$\Pi_{SC}^{c}$	[13,402,992, 29,435,012]
Total	-	-	-	166,190,841	268,902,551	335,802,551	-
$w_{c}=0.25$, $SW_{c}=1,343,510,203$, $CS_{c}=1,007,707,652$							

**Table 6:** Numerical Analysis Results of IBWT Supply Chain Coordination with Ramsey pricing.

i	$w_{ei}^{r}$	$w_{i}^{r}$	$p_{i}^{r}$	$q_{i}^{r}$	$\Pi_{D_{i}}^{r}$	$\Pi_{S}^{r}$	$Range\;of\;w_{ei}^{r}$
1	26,000,000	0.12	0.52	132,602,537	20,139,479	67,635,775	[25,074,215, 26,284,209]
2	35,000,000	0.30	1.09	88,116,831	28,913,858	$\Pi_{LS}^{r}$	[34,733,591, 36,409,712]
3	41,000,000	0.52	1.77	63,661,162	34,149,789	40,581,465	[40,839,689, 42,810,469]
4	46,000,000	0.78	2.58	48,369,034	37,071,339	$\Pi_{ES}^{r}$	[45,144,606, 47,323,126]
5	49,000,000	1.08	3.51	38,002,772	39,948,884	27,054,310	[48,338,722, 50,671,379]
6	51,000,000	1.43	4.58	30,590,805	42,437,213	$\Pi_{SC}^{r}$	[50,777,877, 53,228,239]
Total	-	-	-	401,343,140	202,660,562	270,296,337	-
$w_{r}=0.11$, $SW_{r}=1,622,278,025$, $CS_{r}=1,351,981,687$							

**Table 7:** Sensitivity analysis Results of Water Delivery Loss Rate.

(**a**) Equilibrium without Ramsey Pricing					
**Loss Rate**	**1%**	**2%**	**3%**	**4%**	**5%**
$\Pi_{D_{1}}^{b}$	20,201,177	20,115,690	20,029,550	19,942,747	19,855,271
$\Pi_{D_{2}}^{b}$	28,265,449	28,077,018	27,887,333	27,696,380	27,504,145
$\Pi_{D_{3}}^{b}$	33,547,393	33,248,106	32,947,004	32,644,078	32,339,320
$\Pi_{D_{4}}^{b}$	37,421,213	37,006,456	36,589,363	36,169,944	35,748,212
$\Pi_{D_{5}}^{b}$	40,426,010	39,893,060	39,357,293	38,818,758	38,277,505
$\Pi_{D_{6}}^{b}$	42,839,129	42,186,366	41,530,347	40,871,179	40,208,975
$\Pi_{S}^{b}$	67,466,790	66,742,232	66,013,630	65,281,028	64,544,476
$\Pi_{LS}^{b}$	40,480,074	40,045,339	39,608,178	39,168,617	38,726,686
$\Pi_{ES}^{b}$	26,986,716	26,696,893	26,405,452	26,112,411	25,817,790
$\Pi_{SC}^{b}$	270,167,160	267,268,928	264,354,519	261,424,113	258,477,904
$CS_{b}$	608,101,111	601,580,087	595,022,669	588,429,255	581,800,284
$SW_{b}$	878,268,272	868,849,015	859,377,188	849,853,368	840,278,188
(**b**) Coordination without Ramsey Pricing					
**Loss Rate**	**1%**	**2%**	**3%**	**4%**	**5%**
$\Pi_{D_{1}}^{c}$	27,989,465	27,841,397	27,692,198	27,541,851	27,390,337
$\Pi_{D_{2}}^{c}$	38,957,195	38,630,822	38,302,278	37,971,537	37,638,577
$\Pi_{D_{3}}^{c}$	47,105,789	46,587,409	46,065,884	45,541,201	45,013,346
$\Pi_{D_{4}}^{c}$	52,815,442	52,097,062	51,374,635	50,648,180	49,917,720
$\Pi_{D_{5}}^{c}$	57,019,903	56,096,806	55,168,832	54,236,061	53,298,584
$\Pi_{D_{6}}^{c}$	60,199,548	59,068,929	57,932,671	56,790,958	55,643,987
$\Pi_{S}^{c}$	66,900,000	66,900,000	66,900,000	66,900,000	66,900,000
$\Pi_{LS}^{c}$	40,140,000	40,140,000	40,140,000	40,140,000	40,140,000
$\Pi_{ES}^{c}$	26,760,000	26,760,000	26,760,000	26,760,000	26,760,000
$\Pi_{SC}^{c}$	350,987,340	347,222,425	343,436,498	339,629,789	335,802,551
$CS_{c}$	1,053,262,021	1,041,967,276	1,030,609,494	1,019,189,366	1,007,707,652
$SW_{c}$	1,404,249,361	1,389,189,701	1,374,045,992	1,358,819,155	1,343,510,203
(**c**) Coordination with Ramsey Pricing					
**Loss Rate**	**1%**	**2%**	**3%**	**4%**	**5%**
$\Pi_{D_{1}}^{r}$	20,943,293	20,744,639	20,544,468	20,342,756	20,139,479
$\Pi_{D_{2}}^{r}$	30,682,969	30,245,095	29,804,306	29,360,571	28,913,858
$\Pi_{D_{3}}^{r}$	36,957,096	36,261,616	35,561,918	34,857,982	34,149,789
$\Pi_{D_{4}}^{r}$	40,959,040	39,995,232	39,025,995	38,051,354	37,071,339
$\Pi_{D_{5}}^{r}$	44,941,557	43,703,094	42,458,085	41,206,642	39,948,884
$\Pi_{D_{6}}^{r}$	48,549,139	47,032,255	45,507,806	43,976,037	42,437,213
$\Pi_{S}^{r}$	59,486,762	61,507,227	63,538,969	65,581,864	67,635,775
$\Pi_{LS}^{r}$	35,692,057	36,904,336	38,123,381	39,349,118	40,581,465
$\Pi_{ES}^{r}$	23,794,705	24,602,891	25,415,588	26,232,745	27,054,310
$\Pi_{SC}^{r}$	282,519,857	279,489,159	276,441,546	273,377,205	270,296,337
$CS_{r}$	1,413,099,286	1,397,945,795	1,382,707,732	1,367,386,023	1,351,981,687
$SW_{r}$	1,695,619,143	1,677,434,954	1,659,149,279	1,640,763,227	1,622,278,025

**Table 8:** Sensitivity analysis Results of Ramsey Coefficient (Coordination with Ramsey pricing).

Ramsey Coefficient	0.60	0.65	0.70	0.75	0.80
$\Pi_{D_{1}}^{r}$	20,139,479	18,839,514	17,500,718	16,119,389	14,691,196
$\Pi_{D_{2}}^{r}$	28,913,858	27,113,105	25,258,563	23,345,103	21,366,724
$\Pi_{D_{3}}^{r}$	34,149,789	32,032,468	29,851,901	27,602,058	25,275,885
$\Pi_{D_{4}}^{r}$	37,071,339	34,730,830	32,320,409	29,833,410	27,262,034
$\Pi_{D_{5}}^{r}$	39,948,884	37,442,778	34,861,812	32,198,851	29,445,542
$\Pi_{D_{6}}^{r}$	42,437,213	39,804,649	37,093,450	34,296,116	31,403,877
$\Pi_{S}^{r}$	67,635,775	94,612,830	120,433,944	145,051,268	168,410,948
$\Pi_{LS}^{r}$	40,581,465	56,767,698	72,260,366	87,030,761	101,046,569
$\Pi_{ES}^{r}$	27,054,310	37,845,132	48,173,578	58,020,507	67,364,379
$\Pi_{SC}^{r}$	270,296,337	284,576,173	297,320,797	308,446,195	317,856,206
$CS_{r}$	1,351,981,687	1,313,890,030	1,274,660,559	1,234,184,779	1,192,335,774
$SW_{r}$	1,622,278,025	1,598,466,203	1,571,981,356	1,542,630,974	1,510,191,980
